# Weak anthropogenic electric fields affect honeybee foraging

**DOI:** 10.1016/j.isci.2025.112550

**Published:** 2025-05-19

**Authors:** Victoria J. Mallinson, Fraser A. Woodburn, Liam J. O’Reilly

**Affiliations:** 1 School of Biological Sciences, Life Sciences Building, University of Bristol, 24 Tyndall Avenue, Bristol BS8 1TQ, UK

**Keywords:** Ecology, Entomology, Ethology

## Abstract

Aerial electroreception, the detection of airborne electric fields (E-fields), is an emerging sensory system in arthropods, including bees, which can use floral E-fields as foraging cues. However, the influence of anthropogenic E-fields on these interactions remains underexplored. Through field experiments in urban meadows, we demonstrate that weak anthropogenic E-fields, including alternating current (AC) and direct current (DC) fields, significantly alter honeybee floral landing behaviors. AC and positive DC fields reduced landings by 71% and 53%, respectively, whereas negative DC fields had no statistically significant impact. Measurements of E-fields near high-voltage transmission lines revealed persistent field strengths comparable to those used experimentally, spanning tens of meters at heights relevant for bee foraging. These findings underscore the potential for anthropogenic E-fields to affect and potentially disrupt plant-pollinator interactions, threatening pollination efficiency, a cornerstone of agriculture and biodiversity. Our study highlights the need for advancing research on the ecological impacts of electric pollution.

## Highlights

• Weak anthropogenic-like electric fields reduce honeybee floral landing
• AC and positive DC fields deter honeybee landing, but negative DC does not
• Electric fields near power lines match experimental levels, spanning tens of meters
• These findings highlight electric pollution as a potential disruptor of pollination

## Graphical abstract

**Figure:**
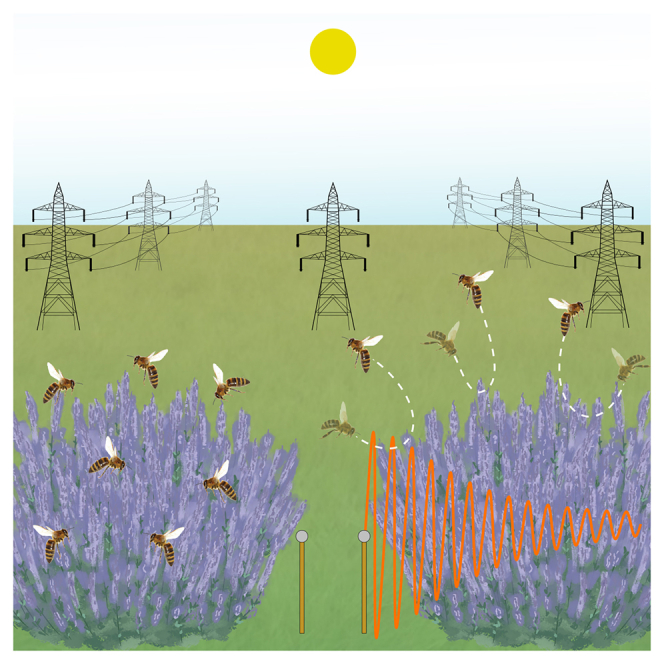

## Introduction

Aerial electroreception, the ability to detect and respond to airborne electric fields (E-fields), is an emerging sensory modality in arthropods.^1^^,^^2^^,^^3^^,^^4^^,^^5^^,^^6^^,^^7^ Empirical and theoretical evidence suggests E-field detection occurs through the sensory multimodality of mechanoreceptors such as hairs and the antennal Johnston’s organ. E-fields exert Coulomb (electrostatic) forces on these mechanoreceptors, stimulating them and activating the same neurophysiological pathways triggered by mechanical stimuli such as touch or airflow.^3^^,^^8^^,^^9^ Behaviorally, spiders can utilize atmospheric E-fields as triggers for dispersal,^5^ caterpillars initiate defensive behaviors in response to E-fields of their predators,^2^ and both hymenopteran and dipteran pollinators may use floral E-fields as foraging cues.^1^^,^^4^ During flight, bees tend to accumulate a positive electrostatic charge,^1^^,^^10^ while flowers are typically negatively charged due to their electrical connection to the Earth and the generally positive atmospheric potential gradient.^11^^,^^12^ These charge dynamics create an electric environment that not only provides foraging cues but also facilitates pollen transfer.^13^^,^^14^

**Figure 1:**
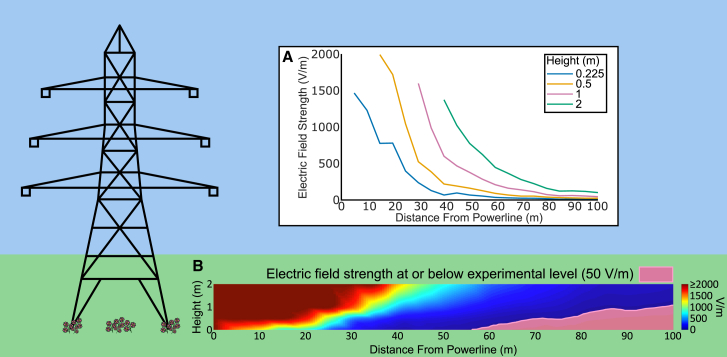
Electric fields produced by 275 kV mains electricity transmission lines in the rural England (A) The electric field strength attributable to the high-voltage AC mains transmission line in horizontal transects measured from directly underneath the cable (0 m) up to 100 m away from the line. Measurements were taken at four different heights between 0.225 and 2 m. The measurement device has an upper range of 2000 V/m, thus, all traces are truncated where closest to the transmission line, as these points registered above this upper limit. (B) The electric field strength of the 2-D area assessed with the vertical and horizontal transects. Linear interpolation has been used to smooth the display. The area highlighted in pink represents the regions measured where the AC electric field was within the range produced during our experimental electric field manipulations (≤50 V/m). Note again that the Z-component (electric field strength) is limited to 2000 V/m.

E-fields encountered by electroreceptive organisms are not exclusively natural. Anthropogenic E-fields have become a pervasive feature of the modern environment.^15^ Sources include power infrastructure, industrial equipment, electrical devices, vehicles, synthetic materials, and household appliances. These fields can overlap in frequency and strength with natural E-fields, potentially interfering with the ability of pollinators to detect floral cues. For instance, power distribution systems generate low-frequency AC E-fields at 50 or 60 Hz,^16^ frequencies that can align with naturally occurring E-field signals such as those produced by insect wingbeats.^17^^,^^18^ High-voltage transmission lines produce alternating current (AC) E-fields persisting for tens of meters into the environment (Figure 1), and can alter the electric environment over a 1 km away in the form of direct current (DC) fields.^19^ Indeed, conservative estimates suggest that in the UK, there is 67,929 km^2^ of land in which bee colonies will be in the foraging range of high-voltage transmission lines,^20^ highlighting the widespread nature of anthropogenic E-field exposure.

The role of pollinators in supporting biodiversity and agriculture underscores the importance of understanding the potential impacts of anthropogenic E-fields. Animal pollination is responsible for approximately 75% of food crops and 35% of global food production, with an estimated annual economic value of $235 to $577 billion.^21^^,^^22^ Pollutants such as light, sound, and chemicals interfere both directly^23^ and indirectly with various animal sensory systems,^24^ including those of pollinators.^25^^,^^26^ Indeed, growing evidence suggests that electromagnetic and electric fields negatively impact various animal taxa again including pollinators.^27^ Low frequency electromagnetic fields produced by power distribution systems affect the cognitive performance of honeybees,^20^ and agrochemicals influence the static electrical properties of flowers, altering bumblebee foraging.^28^

Despite the increasing prevalence of anthropogenic E-fields and their potential to disrupt plant-pollinator interactions, the effects of weak E-fields on pollinator behavior remain poorly understood. Here, we investigated how weak anthropogenic E-fields, comparable in strength to those generated by high-voltage transmission lines, influence the foraging behavior of honeybees (*Apis mellifera*, Linnaeus). We locally altered the E-field around catmint (*Nepeta grandiflora*, Bieb) verticillasters (flower rings) in urban meadows, finding that both weak AC and DC E-fields significantly alter honeybee floral landing.

## Results

### Summary of behavioral observations

**Figure 2:**
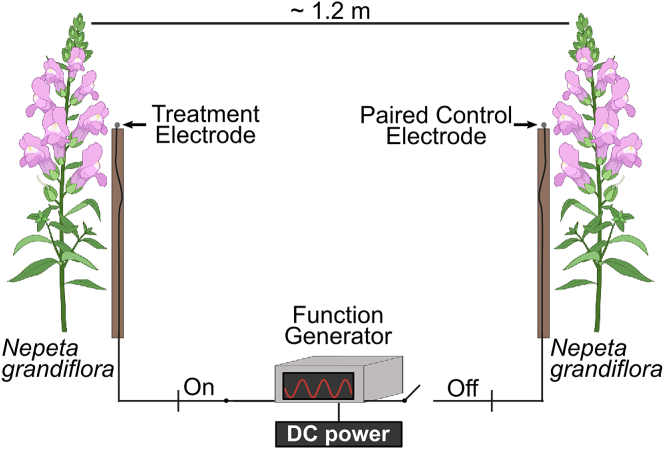
Experimental set-up for electric field manipulation Schematic of the field setup used to investigate the effects of weak anthropogenic electric fields on honeybee (*Apis mellifera*) foraging behavior. Two catmint (*Nepeta grandiflora*) plants, around 1.2 m apart, had electrodes positioned next to them. One flower received an electric field treatment of either AC (50Hz), positive DC, or negative DC, and the Paired Control electrode was electrically floating, providing no electric field. Honeybee landing behavior was simultaneously observed at the two flowers. Flower icons are for illustrative purposes only and not to scale, and do not depict the species used in the experiment. Figure created using BioRender.com.

To explore the effect of weak anthropogenic E-fields on pollinator behavior, field experiments were performed in Bristol, UK. We manipulated the electric environment of flowers with anthropogenic-like E-fields, and observed honeybee landing behaviors. In urban meadows, experimental E-fields local to *Nepeta grandiflora* (catmint) flowers were generated using a portable, battery powered, function generator connected to 1.5 mm diameter spherical silver electrodes positioned 50 mm from the flowers (Figure 2A). Experimental electric manipulations were thus non-contact changes to the E-field local to the flower rather than direct contact changes to the plant’s electric potential. To test the effects of both mains electricity-like AC and static DC anthropogenic E-fields, three electrode potentials were used, 5 Vpp (peak-to-peak voltage) 50 Hz AC, and both positive and negative 5 V DC. Herein, these treatments will be referred to as AC, positive DC, and negative DC, respectively.

Honeybee foraging behavior, specifically floral landing events, was observed simultaneously at two flowers, an experimental treatment flower and a Paired Control flower (Figure 2). The Paired Control flower was treated identically to the treatment flower, other that its electrode did not produce a stimulus E-field and was electrically floating (Figure 2). Paired Controls ensured that honeybee behaviors in response to the E-field stimulus could always be assessed relative to a control under similar spatial and temporal conditions. Each of the three experimental set-ups was observed for 2 h, eight times (trials). For each trial, different plants were observed, and no plants were ever used more than once.

### Alternating current and direct current effects on honeybee-flower interactions

**Figure 3:**
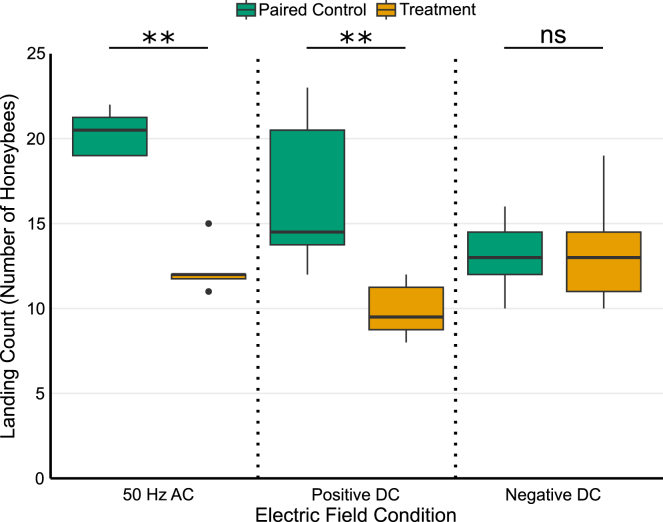
Impact of electric field treatments on honeybee landing Boxplot displaying the landing counts of honeybees (*Apis mellifera*) on *Nepeta grandiflora* (catmint) under three electric field treatments. From left to right: AC (50 Hz), positive DC, and negative DC, with each treatment accompanied by the landing counts of a Paired Control (*n* = 8 for all treatments and controls). Whiskers extend to the minimum and maximum data points within 1.5 times the interquartile range (IQR), while horizontal lines through each box indicate the median, and dots represent outliers. Asterisks denote significance from paired permutation tests: ns (non-signficant), *p* ≥ 0.05, ∗*p* < 0.05, ∗∗*p* < 0.01, and ∗∗∗*p* < 0.001.

When E-fields were altered experimentally, the median number of bees landing on the flowers (landing counts) was reduced by 70.8% and 52.6% at the flowers receiving AC and positive DC treatments, respectively, compared to the Paired Control flower (Figure 3). In contrast, no difference in landing counts was observed for the negative DC treatment (0% change; Figure 3).

Paired permutation tests revealed significant reductions in landing counts for AC (observed difference = 8.25, *p* = 0.0084) and positive DC (observed difference = 6.75, *p* = 0.0084) treatments. The null distributions had means near zero (−0.03 and −0.01) and standard deviations of 3.01 and 2.57, respectively, with 95% confidence intervals of [-5.7500, 6.0000] for AC and [-4.7500, 4.7500] for positive DC. The effect sizes, as Cohen’s d, for both were large (2.74 and 2.62, respectively). However, for the negative DC treatment, there was no significant difference in landing counts between the treatment and the Paired Control flower. The observed difference was −0.63 (*p* = 0.5303), and the null distribution mean was near zero (−0.01) with a standard deviation of 0.72. The 95% confidence interval was [–1.38, 1.38], and the Cohen’s d effect size was −0.86. Details on the statistical methods used are provided in the STAR Methods section.

**Figure 4:**
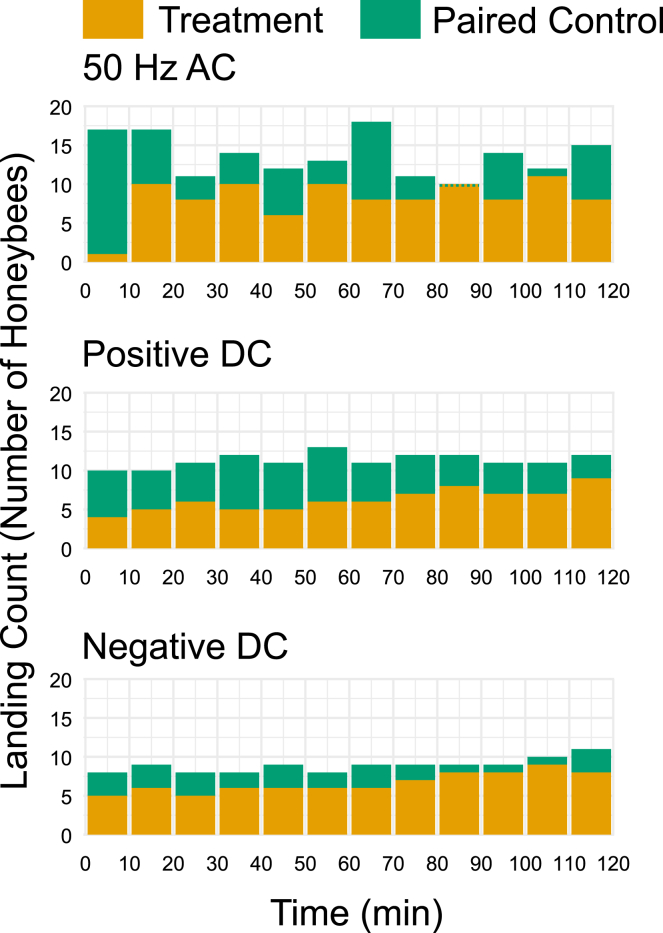
Time-resolved analysis of honeybee landing behavior under electric field treatments Temporal histograms showing pooled honeybee (*Apis mellifera*) landing counts for three electric field treatments. From top to bottom: AC (50 Hz), positive DC, and negative DC, with each treatment accompanied by the landing counts of a Paired Control. Data are grouped into ten-min time bins and include all experimental repeats (*n* = 8 for each treatment and associated Paired Control), these data represent pooled raw counts. A green dotted line represents the value of the Paired Control if it is equal to or less than the treatment and thus obscured.

In order to assess the temporal variation in the observed behaviors over the experimental period, landing counts were pooled from all eight repeats for each treatment and binned into ten-minute windows (Figure 4). During the first ten minutes of the AC treatment, landing counts at the treatment flower were 5.9% of those at the Paired Control landing. Landing counts increased to 58.8% of the Paired Control during the next ten-minute interval. For the remainder of the trial, the percentage fluctuated between 44.4% and 100%, with a mean of 68.1% (±17.4%, SD).

For the positive DC treatment, landing counts at the treatment flower increased gradually over time, starting at 40.0% of the Paired Control and reaching 75.0% (55.0%, mean; ± 10.8%, SD) by the end of the trial, with a mean rate of increase of 0.32% per minute (±0.66% per minute, SD; Figure 4). In contrast, during the negative DC treatment, landing counts at the treatment flower remained more consistent with the Paired Control throughout (74.4%, mean; ± 10.2%, SD), gradually increasing from 62.5% to a peak of 90% of the Paired Control during the first 110 min before declining slightly to 72.7% during the final 10 min. The mean rate of change during the first 110 min was 0.28% per minute (±0.76% per minute, SD), and 0.09% per minute (±0.92% per minute, SD; Figure 4) over the entire trial.

### Quantification of electric fields

To characterize the E-fields used experimentally, measurements were made in the laboratory with the same equipment used to generate the stimuli in the field. The reasons for making measurements in the laboratory instead of in the field were two-fold, firstly, it was then possible to control the electric environment with noise solely originating from mains power, and secondly, it made incremental distance measurements more accurate than in the field. E-field measurements were taken at 70 mm and 400 mm from potted *Nepeta grandiflora* flowers using 1.5 mm spherical silver electrodes connected to custom-built electrometers (see STAR Methods for the calibration procedure), with a third, identical electrode placed 50 mm from the flower used to produce the stimulus E-field. This stimulus set-up was identical to that used in the field set-up. Here, however, 20 Hz was used as the stimulus frequency, and a 30 Hz lowpass filter was applied to recordings to isolate the stimulus signal from 50 Hz mains electricity noise.

Recordings of E-fields were made under three different conditions, with a *Nepeta grandiflora* present and electrically floating (representing a plant with poor electrical connection to earth in the field, analogous to a drought situation), with the plant present and grounded (representing a plant with a strong electrical connection to earth), and with the plant absent to allow us to assess the impact of the plant on the undisturbed E-field (Table 1).

**Table 1:** Experimental treatment conditions for measuring electric field (E-field) strengths around *Nepeta grandiflora* flowers in the laboratory

Condition	Description
Grounded Plant	The plant was present and electrically grounded by a wired connection to the water-saturated potting compost.
Floating Plant	The plant was present, but the potting compost was floating (not electrically grounded).
No Plant	The plant and pot were entirely removed from the setup, while all other equipment remained.

The conditions were designed to assess the impact of plant presence and electrical grounding on E-field distribution.

**Figure 5:**
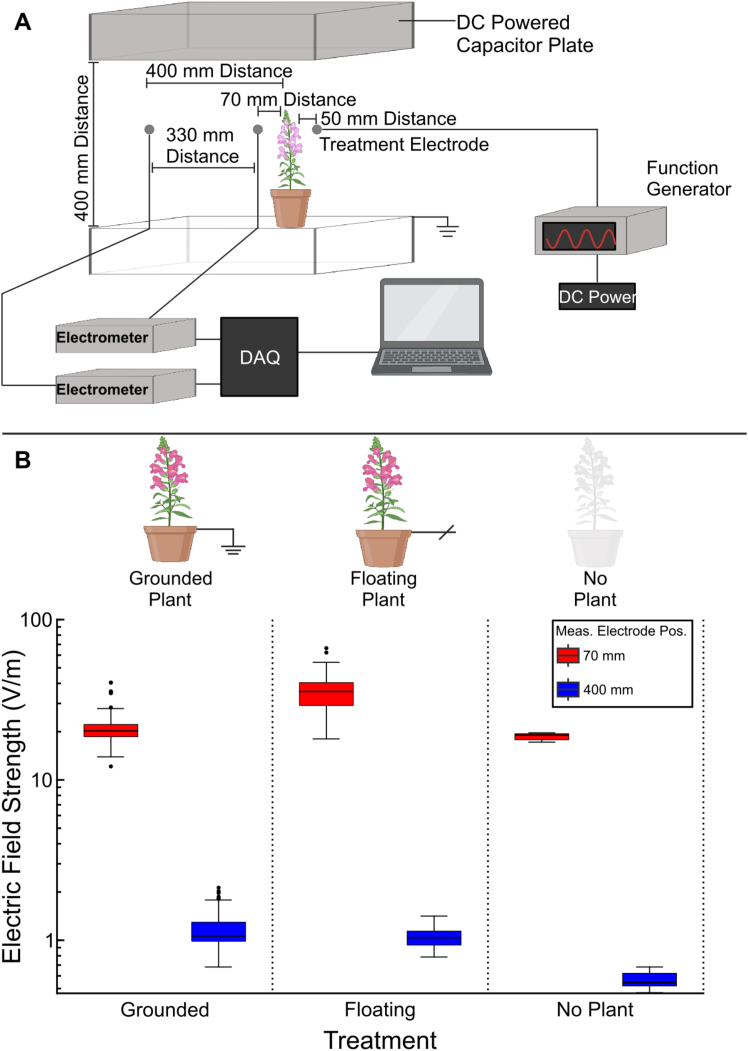
Laboratory setup and electric field strength measurements (A) A schematic of the laboratory set-up used to measure experimental electric field strengths. (B) Boxplots representing electric field strengths at two distances (70 and 400 mm) from a 1.5 mm diameter spherical silver stimulus electrode, as shown in panel A, powered with a 5 V pp 20 Hz sine wave. Whiskers extend to the minimum and maximum data points within 1.5 times the interquartile range (IQR), while horizontal lines through each box indicate the median, and dots represent outliers. The influence of plant presence on electric field strength was tested with three treatments. Electric field strengths are displayed on a logarithmic scale. Partially created with Biorender.com. See also Figures S1–S3.

Measured E-fields ranged from 12.14 to 66.36 and 0.47 to 2.13 V/m for the 70 and 400 mm measurements, respectively, across all conditions (Figure 5). E-fields measured at 70 mm were 21.99 ± 6.36, 34.60 ± 11.11, and 16.99 ± 0.93 V/m, and at 400 mm were 1.20 ± 0.37, 1.04 ± 0.14, and 0.56 ± 0.06 V/m (mean ± standard deviation) for the Grounded Plant, Floating Plant, and No Plant conditions, respectively. The E-field strength at 400 mm increased with the presence of a plant, whether electrically grounded or floating, and E-field strength at 70 mm was increased when the plant was floating compared to being grounded or absent (Figure 5).

### Anthropogenic electric field measurements

To contextualize the E-fields produced in our experiments, we measured the extent to which electric fields produced by high-voltage mains electricity transmission lines extend through the environment at heights relevant to foraging bees (0.225–2 m). In rural England, we took horizontal transect measurements of E-fields from directly underneath a set of 275 kV transmission lines (0 m) to 100 m from the source using an AC-sensitive E-field meter in five meter increments at four different vertical heights (see STAR Methods for full details). Unsurprisingly, E-field strength was highest in closest proximity to the transmission lines and attenuated with distance (Figure 1). Interestingly, the E-fields persisted in strengths above or within a similar range to those used in our experiments across the full range of both horizontal and vertical transects (Figure 1B). Directly under the transmission line, E-field strengths at all heights were above the upper limit of the measurement device (2000 V/m), and these overrange values persisted further horizontally the higher the measurement device was positioned (Figure 1A).

## Discussion

Here, we demonstrate that weak localized anthropogenic E-fields, comparable in strength to those measured tens of meters away from high-voltage transmission lines, alter honeybee foraging behavior. Honeybees exhibited reduced landing in response to both mains electricity-like AC (50 Hz) and positive DC E-fields. By contrast, negative DC fields had no measurable impact on landing behavior (Figure 3). Notably, we avoided any physical modification of flowers during our experiments, with only electrodes placed in their vicinity, avoiding contact, in order to alter their immediate electrical environment. These findings demonstrate that pollinators are responsive to even weak changes in the electrical environment surrounding flowers. It is therefore conceivable that anthropogenic E-fields, such as those produced by high-voltage transmission lines, could influence the foraging behavior of pollinators and their interactions with flowers.

Intriguingly, although both AC and positive DC E-fields reduced the occurrence of landing, negative DC fields did not alter landing counts relative to their Paired Control (Figure 3). These contrasting responses may reflect how honeybees interpret different electrical cues in their environment, relating to their natural electric ecology. Naturally occurring floral E-fields are likely to be negative due to generally positive atmospheric E-fields and the electrical connection of plants to the earth.^6^^,^^12^ Thus, the negative DC experimental E-fields may offer little disturbance, or even enhance the expected negative charge offered by plants and their floral structures. In contrast, positive DC fields can be seen as reducing the attractiveness of the flower as a foraging target, as they could suggest prior visitation by another pollinator. Pollinator landing alters the electric potential, and thus the E-field, of plants for tens of seconds, sometimes as much as minutes.^1^

AC E-fields, on the other hand, are temporally dynamic, oscillating between positive and negative polarity. Such oscillations represent a stimulus rarely, if ever, encountered in natural contexts, potentially explaining their more pronounced deterrent effect (Figures 3 and 4). Whilst natural oscillating E-fields at frequencies similar to mains electricity may be encountered, such as those generated by insect wingbeats,^17^^,^^18^ they fundamentally differ as they do not switch polarity. Instead, these fields are represented as oscillations superimposed on a significant DC offset attributable to the insect’s bulk charge (either positive or negative). The observed responses align with the concept that electric cues are integral to honeybee decision-making processes during foraging, and that disturbances to this electric interaction could disrupt pollination efficiency.

Pronounced temporal effects are also revealed, particularly during the AC treatment, where landing was greatly reduced during the first 10 min of the E-field onset (5.9% of the Paired Control), followed by a rapid increase in landing within the next 10 min (58.8% of the Paired Control), and fluctuating thereafter between 44.4% and 100% of the Paired Control without consistently reaching control levels (Figure 4). Similarly, landing increased over time during the positive DC treatment (40%–75% of the Paired Control), but this was gradual throughout the trial period (0.32% per minute), not showing a rapid increase such as the AC treatment (Figure 4). There was also an increase in landing over time during the negative DC treatment, starting at 62.5% and peaking at 90% of the Paired Control, but it differed less from the Paired Control than the positive DC treatment (means of 74.4% and 55.0% respectively; Figure 4). These patterns could suggest that novelty plays a role in the behavioral response of the bees, as the unfamiliar E-fields initially deterred landing. The increase in landing over time could potentially be the result of sensory adaptation or learning. It is well documented that honeybees learn about foraging sources and locations.^29^^,^^30^^,^^31^ To test the presence of learning or adaptation, similar experiments could be run, including the identification of individual bees the evaluate performance in time, something beyond the scope of the current study. The patterns observed here suggest that plant-pollinator interactions may show some resilience to electrical disturbance, and the nature of the observed plasticity of honeybee behavior warrants further exploration.

A potential mechanism underlying these behavioral changes could be neophobia, the avoidance of novel situations and stimuli.^32^ Indeed, honeybees will avoid artificial flowers with new colors despite increased floral reward^33^ and bumblebees exhibit a lag in acceptance time of novel flowers.^34^ Although we did not directly test for neophobia in this study, the reduced landing rates during the initial stages of exposure to AC and positive DC fields may suggest a similar hesitancy toward novel E-field conditions. Over time, however, bees might overcome this hesitation through repeated exposure. Further research is needed to determine whether such adaptation occurs consistently across natural and anthropogenic contexts and E-field intensities.

Further complicating this interpretation is the fact that the E-fields produced in our experiments were consistent in strength and dynamics over the experimental period, providing a stable stimulus that could allow for sensory adaptation or favor learning. In contrast, anthropogenic E-fields, such as those produced by high-voltage transmission lines, often exhibit significant variability in field strength due to seasonal, daily, and even hourly fluctuations in power grid loads.^35^ Additionally, DC E-field temporal variability can be caused by corona ions from high-voltage transmission lines,^36^ as well as other sources of anthropogenic disturbance, such as daily vehicle emissions increasing during rush hour.^37^ This variability could present a more complex and unpredictable challenge for foraging pollinators, potentially limiting their ability to adapt or habituate to anthropogenic electric disturbances. Investigating behavioral responses to dynamic E-fields would be a critical next step in understanding the real-world implications of the present findings.

Importantly, the observed honeybee behaviors may not be a direct response to sensing the experimental E-fields and could be mediated by floral cues. The electric ecology of plant-pollinator interactions is complex, actively involving both parties. Flowers act as electrical antennas,^38^ with some plants even increasing volatile production in response to the E-fields of bees.^39^ So, several non-exclusive scenarios become apparent, whereby bees react directly to the experimental E-fields, indirectly in response to changes in floral cues, electric or otherwise, or a combination of the above. The exact mechanisms underlying the behaviors are beyond the phenomenology unveiled in this study and remain unclear, however, the behaviors themselves are apparent and are concerningly triggered by weak E-fields.

Unfortunately, our findings contribute to the ever-growing body of literature demonstrating the extent of human impact on the environment and, in particular, pollinators. The reduction in honeybee landing counts observed in response to anthropogenic E-fields complements similar findings in bumblebees.^28^ Detrimental effects have also been observed in honeybee hives placed below high-voltage transmission lines, but the incident E-fields at play in that situation were over 100 times greater than those we produced here.^40^ Indeed, the fields we measured emanating from high-voltage transmission lines were far greater than our experimental E-fields (Figures 1 and S2). Notably, the strength of the experimental E-fields used here and by Hunting et al.^28^ are low; we produced fields of around 10–60 V/m at 70 mm from the electrode (Figure 3), and the fields Hunting et al. produced at the same distance from the flower were around 250 V/m. The appreciation of the quantities involved highlights the sensitivity of pollinators to even minor alterations in their electrical environment, emphasizing the importance of understanding the broader ecological impacts of anthropogenic E-fields.

This study demonstrates that even weak anthropogenic electric fields can significantly reduce floral landing by the honeybee (*Apis mellifera*), a key pollinator. While focused on a single plant-pollinator interaction, these findings suggest that low-intensity electric pollution may disrupt natural pollinator behaviors, warranting further investigation. Addressing this overlooked aspect of ecological interactions represents a critical frontier for multidisciplinary research, helping to quantify the broader ecological consequences of electric pollution.

This study provides important insights into the impacts of anthropogenic E-fields on a plant-pollinator interactions, but it also highlights several areas for future research. Our study focused on the short-term effects on a single plant-pollinator interaction, and although similar results were found with bumblebees and lavender flowers,^28^ generalization of our findings must be considered carefully. Thus, investigations into a broader range of pollinator and plant species, including pollination networks, as well as longer-term impacts are key to exploring the influence of electric pollution. Additionally, further work is needed to explore the physiological mechanisms of both bees and plants underpinning behavioral responses. It would also be pertinent to study foraging in natural environments with strong and variable anthropogenic E-fields, such as those near to power infrastructure, as well as manipulating electrically pristine environments with E-fields mimicking high-voltage transmission lines. This study should act as a foundation for future research into an understudied form of global pollution and its impact on electric ecology, and, in particular, beneficial electroreceptive and electrosensitive organisms.

In summary, this study demonstrates that even weak anthropogenic electric fields can significantly reduce floral landing by the honeybee (*A. mellifera*), a key pollinator. Although focused on a single plant-pollinator interaction, these findings suggest that low-intensity electric pollution may disrupt natural pollinator behaviors, calling for further investigation into this underappreciated facet of electric ecology. These findings highlight a critical frontier for multidisciplinary research, addressing the largely unquantified extent to which electric pollution pervades ecological processes and interactions.

### Limitations of the study

Our study demonstrates that weak anthropogenic E-fields significantly alter honeybee foraging behavior, with AC and positive DC fields reducing floral landings while negative DC fields have no effect, providing strong evidence that even subtle changes in the local electric environment can influence pollinator decision-making. However, our findings focus on short-term effects within a single plant-pollinator interaction, and further research is needed to assess how these responses generalize across different pollinators, plant species, and broader ecological networks. The long-term consequences of anthropogenic E-field exposure on pollination dynamics remain unknown, as do the physiological and behavioral mechanisms underpinning these effects. Additionally, our findings are based on the experimental alteration of local floral E-fields, and future research should explore how electric pollution affects plant-pollinator interactions in natural environments, particularly near power infrastructure where anthropogenic E-fields are strong and variable.

## Resource availability

### Lead contact

Further information and requests for resources and reagents should be directed to and will be fulfilled by the lead contact, Liam O’Reilly, liam.oreilly@bristol.ac.uk.

### Materials availability

This study did not generate new unique reagents.

### Data and code availability

• All data have been deposited at Mendeley Data and are publicly available as of the date of publication. Accession numbers are listed in the key resources table DOI is listed in the key resources table.
• This article does not report original code.
• Any additional information required to reanalyze the data reported in this article is available from the lead contact upon request.

## Acknowledgments

RESURG: Research Experience for Students from Under-Represented Groups awarded to LO alongside Isaac Chenchiah, Ryan Palmer, Mark Schenk, Benjamin King Sutton Woods, Matt O’Donnell, Rainer Groh, and Rachel Bennett by the 10.13039/501100000883University of Bristol, supplied by Research England (RE-CL-2022-06) supported VM, the 10.13039/501100000781European Research Council (ERC-ADG 743093 ELECTROBEE) awarded to Prof. Daniel Robert supported LO and VM, and 10.13039/501100000268BBSRC (Grant BB/T003235/1) also awarded to Daniel Robert supported LO. A 10.13039/501100000268BBSRC SWBio Center for Doctoral Training studentship awarded to Daniel Robert supported FW. We thank Daniel Robert for his invaluable advice and guidance, and the Electric Ecology lab group for their support and insightful discussions. We also thank Prof. Giles Harrison for his guidance and expert advice on electrometer design and construction. We thank all the contributors to the RESURG program 2022.

## Author contributions

LO conceived the study, secured funding for VM, and provided critical revisions to the article. VM collected and analyzed the behavioral and laboratory electric field data, and prepared the initial article draft. FW collected electric field measurements of transmission lines. LO and VM collaboratively refined the study’s objectives and contributed significantly to the final article, with LO leading the article’s revision process.

## Declaration of interests

The authors declare no competing interests.

## STAR★Methods

### Key resources table

**Table:** 

REAGENT or RESOURCE	SOURCE	IDENTIFIER
**Deposited data**		

Honeybee Visitation Analysis	This paper	Mendeley Data: https://doi.org/10.17632/wrwdwy3x36.1
Laboratory Electric Field Recordings	This paper	Mendeley Data: https://doi.org/10.17632/p5znz78ttw.1

**Software and algorithms**		

MATLAB 2018b	MathWorks, Portola Valley, California, United States	https://www.mathworks.com/
R 4.3.2	R Foundation for Statistical Computing, Vienna, Austria.	https://www.r-project.org/
RStudio	Posit PBC, Massachusetts, US	https://posit.co/

**Other**		

FG-100 DDS Function Generator	Unknown Manufacturer	N/A
USB-6009 DAQ	National Instruments, Austin, Texas, United States	https://www.ni.com/en.html
Metrix BioTest VX0100 Electric Field Strength Meter	Chauvin Arnoux UK Ltd, Dewsbury, UK	https://www.chauvin-arnoux.co.uk/en
PS350 High Voltage Power Supply	Stanford Research Systems, Sunnyvale, California, USA	https://www.thinksrs.com/

### Experimental model and study participant details

#### Honeybees and flowers

Honeybees (*Apis mellifera*) were observed foraging on Caucasus catmint (*Nepeta grandiflora*) at two urban meadow locations in Bristol, UK (Royal Fort Gardens, 51.4583 °N, 2.6035 °W, and Brandon Hill Park, 51.45281 °N, -2.6068 °W). Both field sites were visited for experiments between July and September 2023.

### Method details

#### Species and field locations

We chose to investigate how floral visitation behaviour of pollinators in urban meadows was influenced by anthropogenic E-fields. *Nepeta grandiflora* (Caucasus catmint) was selected to have its local electric environment altered with anthropogenic E-field treatments, and *Apis mellifera* (honeybee) was selected as the pollinator species of interest. The plants were identified using the PlantNet app and PlantNet identification webpage.^41^

The PlantNet app was used in the field to take photographs of the *N. grandiflora* plants, which were then analysed using its automated image recognition feature to suggest potential species matches based on plant morphology. This initial identification was further cross-referenced and verified using the PlantNet identification webpage, which provides detailed visual and morphological descriptions for the plant species. *Nepeta grandiflora* was selected for this study as they were common and numerous across our two urban meadow field sites, were frequently visited by honeybees, and have a long field flowering season.^42^

#### Behavioural observations

In all experiments, two plants were observed simultaneously: one received the treatment, while the other served as a Paired Control, equipped identically but without any electrical stimulus applied. During pilot data collection, it was determined that even during high levels of pollinator visitation one observer could easily keep track of the activity at two plants simultaneously, thus, we were able to use the same observer for all treatment flowers and for all trials throughout the experimental period.

Two 1.5 mm diameter spherical silver electrodes were soldered to 3 m long coaxial cables and mounted onto 1 m pieces of bamboo. Each electrode was positioned 50 mm away from an individual *N. grandiflora* verticillaster (flower ring) by inserting the bamboo into the earth close to the stem. Plants selected for each observation period met specific criteria: they were positioned at 1.2 meters (± 0.1 metres) from each other, were attached to different plants rather than being stems on the same plant, had similar heights, and were located away from meadow edges and shaded areas. These selection criteria ensured consistent stimulus exposure conditions and minimised external environmental variation. In each observation period, one electrode was connected to a signal generator (Battery operated ‘FG-100 DDS Function Generator’, Unknown Manufacturer) outputting the E-field stimulus, and the other electrode was electrically floating (i.e., was not held at an electric potential or grounded) to act as a Paired Control (Figure 2A; Table 1). This design provided temporally and spatially matched controls for comparison.

Before each observation period, the two *N. grandiflora* subjects were left for fifteen minutes for wild pollinators to acclimatise to the presence of the setup. Pilot trials showed pollinators resumed normal foraging within 15 minutes of setup disturbance, enabling us to standardise a 15-minute acclimatisation period for the experiment. Following this fixed acclimatisation period, the treatment signal was applied, and observations of honeybee visitations began. Observations were made by one observer (the same observer for all experiments) over a 120-minute period for each treatment, with the observer positioned equidistant (at 1.2 m away) between the treatment flower and the Paired Control flower from the beginning of the acclimatisation period (135 minutes in total). If a honeybee landed on the focal flowers, a count and time of visitation to the closest minute were taken.

#### Treatments

The influence of three types of anthropogenic-like E-fields were investigated, mains electricity-like AC, positive DC, and negative DC. The signals applied to the spherical electrodes were 50 Hz 5 Vpp (peak-to-peak voltage), +5 V DC, and -5 V DC respectively, with every repeat including a Paired Control. Every treatment was repeated eight times, thus, for each E-field treatment a total of 16 flowers were used, resulting in a total of 48 *N. grandiflora* plants over the entire experiment.

We conducted experiments over one foraging season but split into two discrete sampling periods. Foraging conditions for honeybees differ throughout the annual foraging period,^43^ however, through a paired experimental design we aimed to mitigate any influence this had on our data. Trials involving E-field manipulation took place on the following dates: the positive DC signal treatment (*n* = 3) from 07/07/23 to 19/07/23, the negative DC signal treatment (*n* = 2) from 07/07/23 to 19/07/23, the AC treatment (*n* = 8) from 20/07/23 to 25/07/23, the positive DC signal treatment (*n* = 6) from 22/09/23 to 28/09/23, and the negative DC signal treatment (*n* = 5) from 23/09/23 to 30/09/23.

#### Laboratory E-field measurements

To determine the E-field strengths generated by the stimulus electrodes in our field experiments, we used the same equipment from the field experiments to generate E-fields around potted *Nepeta grandiflora* flowers in controlled laboratory conditions. E-field measurements were made in the laboratory as opposed to *in situ* in the field primarily as this allowed for better control of extraneous electric fields not produced by our set-up, but also facilitated distance transect measurements of the field strength.

Laboratory measurements were conducted using potted *Nepeta grandiflora* plants grown under average conditions of 21.0°C (min 19.5°C, max 23.1°C) and 71.0% relative humidity (min 44%, max 97%) in the GroDome of the Life Sciences Building, University of Bristol, UK. E-fields were recorded with two custom-built electrometers^44^ each connected to a 1.5 mm spherical silver electrode. The recording electrodes were positioned inside a polycarbonate box (1000 mm x 750 mm x 400 mm) with a 330 mm separation distance. A potted *N. grandiflora* was placed at a fixed location within the box, situated 70 mm from one recording electrode and 400 mm from the other to simulate distances relevant to a foraging pollinator.^45^ A third 1.5 mm spherical silver electrode for E-field stimulus delivery was positioned 50 mm from the flower ring, replicating the field setup (Figure 5A).

To simulate the atmospheric potential gradient (APG) on a fair-weather day (∼150 V/m^11^), a parallel capacitor was created using a 900 mm x 630 mm steel plate on top of the box at a fixed DC potential of 60 V using a high-voltage power supply (PS350; Stanford Research Systems, Sunnyvale, CA, USA). A ground plate was positioned 400 mm from the top plate, covering the entire base of the box and earthed to the building ground, creating a setup analogous to the ionosphere-Earth charge separation. However, ironically due to the experiments being conducted in a building with extensive mains electricity, 50 Hz electrical noise was recorded by our measurement system. To mitigate this, the stimulus frequency was lowered to 20 Hz (sinewave frequency should not influence the E-field strength), and a low pass filter at 30 Hz was applied to the recorded signals to isolate the stimulus E-field from electrical noise.

Three experimental treatments were conducted (Table 1). The ‘Electrically Floating Plant Present’ treatment consisted of the plant being present in the setup but not electrically connected top ground (number of individual plants, *n* = 5). In the ‘Grounded Plant Present’ treatment, the plant was present and grounded via a wired connection to the water-saturated potting compost (*n* = 5). In the ‘No Plant’ treatment, the plant was entirely removed from the setup (*n* = 5). For each treatment, electrometer recordings were obtained using a custom MATLAB script (Version 2018b, MathWorks, Portola Valley, California, USA), interfaced with a USB-6009 DAQ (National Instruments, Austin, Texas, USA).

#### Transmission line electric field measurements

A 275 kV high-voltage mains electricity transmission line located in a rural environment in South West England was selected for measurement. E-fields were measured using a Metrix BioTest VX0100 hand-held Electric Field Strength Meter (Chauvin Arnoux UK Ltd, Dewsbury, UK) electrically grounded by the user through the metal finger pad on the back of the device. To assess an area relevant for pollinator foraging, horizontal transects of 0 (directly under the transmission line) to 100 m perpendicular to the transmission line were made at four different vertical heights; 0.225 m (the lowest height possible given the instrument’s dimensions), 0.5 m, 1 m, and 2 m. Measurements were taken in five metre increments along these horizontal transects. This allowed us to generate a two-dimensional map of the electric field strength emanating from the transmission line (Figure 1B). Note the E-field meter has an upper range of 2000 V/m which was exceeded when close to the transmission line, which meant exact measurements of the E-field in close proximity were not possible.

### Quantification and statistical analysis

#### Behavioural observation analysis

All statistical tests were performed using R^46^ (Version 4.3.2) in R Studio.^47^ The behavioural datasets were first tested for normality and homogeneity of variance using the Shapiro-Wilks test using the stats package^46^ and Levene’s test using the car package,^48^ respectively. Paired permutation tests, using the dplyr package^49^ were used to compare groups, generating null distributions from 10,000 random label permutations. Statistical significance (p<0.05) was determined by the proportion of permuted differences matching or exceeding the observed difference. Effect sizes (Cohen’s d) and 95% confidence intervals were also calculated. For these experiments the sample size (*n*) represents the number of repeats of the two-hour observation periods of a treatment and paired control flower.

A time-resolved analysis was performed to assess the data for temporal effects over the experiment duration. For each treatment, data from all eight repeats were pooled and placed into 10-minute time bins (0-9.99, 10-19.99, 20-29.99, 30-39.99, 40-49.99, 50-59.99, 60-69.99, 70-79.99, 80-89.99, 90-99.99, 100-109.99, and 110-120 mins). The data for each set of treatments and Paired Controls were then plotted as temporal histograms (Figure 4).

#### E-field quantification & electrometer calibration

We calibrated our two custom-built electrometers due to differences in absolute measurement values between them as well as the fact that they measure electric potential and not electric field strength directly. To do so, we firstly measured the electric field strength at distances of 60-600 mm from the stimulus electrode in 60 mm increments using a Metrix BioTest VX0100 Electric Field Strength Meter (Chauvin Arnoux UK Ltd, Dewsbury, UK). As this instrument measures the total alternating current (AC) E-field strength and the room was contaminated with 50 Hz mains-derived E-fields, we also took control measurements without powering the stimulus electrode. One set of measurements were taken with the electrode electrically floating and another with it grounded, as well as measurements with no electrode present (Figure S1). These results demonstrated that some of the measured E-field strength was attributable to the extraneous AC E-fields (probably 50 Hz), and that when the electrode is floating it seems to act as an emitting antenna, amplifying the strength of these fields. As such, for a more accurate measurement of solely our 20 Hz test signal, we subtracted the grounded electrode measurements from those of the test stimulus. We also modelled the root mean square (RMS) electric field strength at the same distances, assuming the 1.5 mm diameter spherical silver electrode acted as a point source. The E-field it produced attenuated with an inverse relationship to distance, consistent with the following equation:

(Equation 1) $$E_{RMS}=\frac{V_{RMS}}{r}$$

where:

*V*_*RMS*_ is the root mean square value of the applied 20 Hz sinewave,

*r* is the distance from the centre of the spherical silver electrode.

When comparing the measured and modelled E-field strength there is not a linear relationship between the two sets of values (Figures S2A and S2B), however, if the data are divided into near (60-300 mm) and far (360-600 mm) distances, then the relationships between the two datasets show a strong positive linear correlation (Figures S2C and S2D). We used these two regression line equations (Figures S2C and S2D) and E-field strengths calculated from Equation 1 above to calculate what would have been the measured E-field strength at the location of the two measurement electrodes connected to the electrometers (70 mm – 18.61 V/m; and 400 mm – 0.56 V/m). Using these values we then determined a calibration factor for each electrometer’s measurements by dividing these E-field strengths by the mean electric potential measured at 70 mm and 400 mm when no plant was present. The calibration factor was then applied to the measurements taken in all the laboratory experiments to calculate the measured E-field strength.
