# Supplementary material for: Weak anthropogenic electric fields affect honeybee foraging
Source: iScience. 2025 May 19;28(6):112550. doi: 10.1016/j.isci.2025.112550 (PMC12225925; doi:10.1016/j.isci.2025.112550)
Supplement: Document S1. Figures S1–S3 [file mmc1.pdf]

iScience, Volume *28*

Supplemental information

Weak anthropogenic electric fields affect honeybee foraging

Victoria J. Mallinson, Fraser A. Woodburn, and Liam J. O'Reilly

Supplementary material

Electric field calibrations

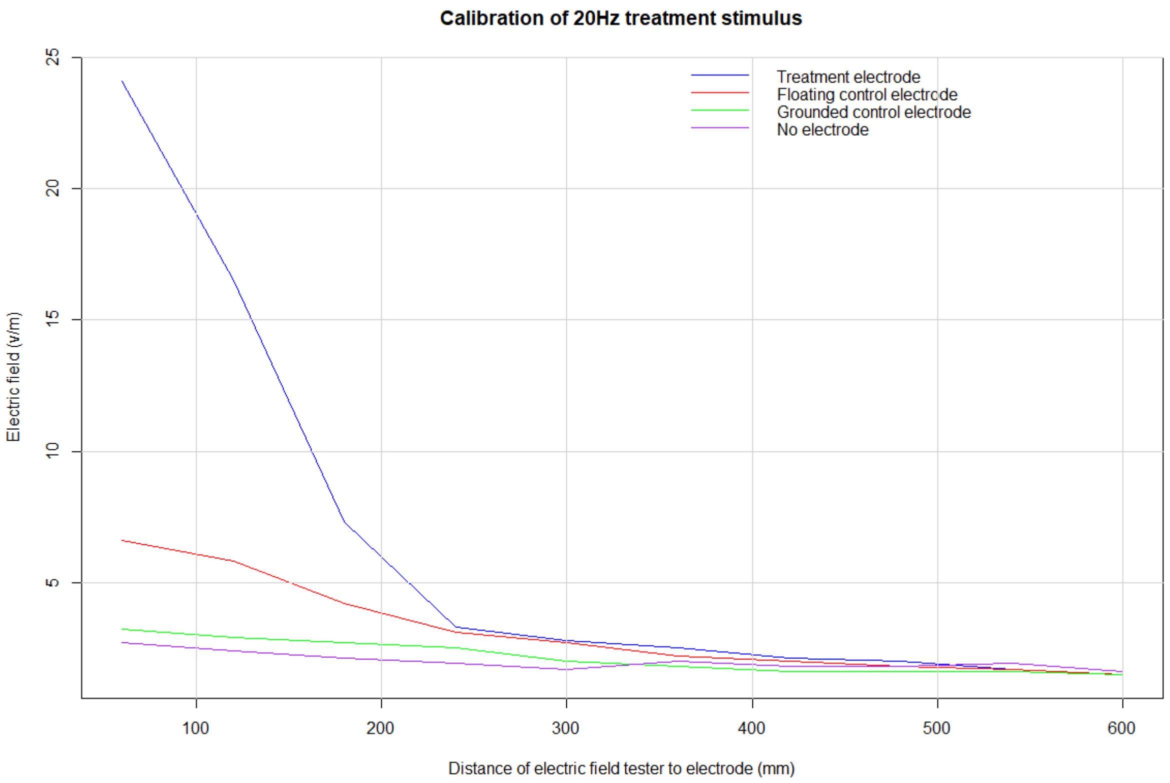

Figure S1. Electric fields recorded from the treatment electrode in the laboratory experiments, related to Figure 5. Plot showing the electric fields (V/m) recorded using a handheld electric field tester (Metrix BioTest VX0100 Electric Field Strength Meter) at distances of 60mm to 600mm away from the treatment electrode within the Perspex box. E-fields were recorded when the 20 Hz 5 V pp was outputted from the function generator, when the signal was turned off and the treatment electrode was electrically floating, when the signal was turned off and the treatment electrode was electrically grounded, and when the treatment electrode was removed.

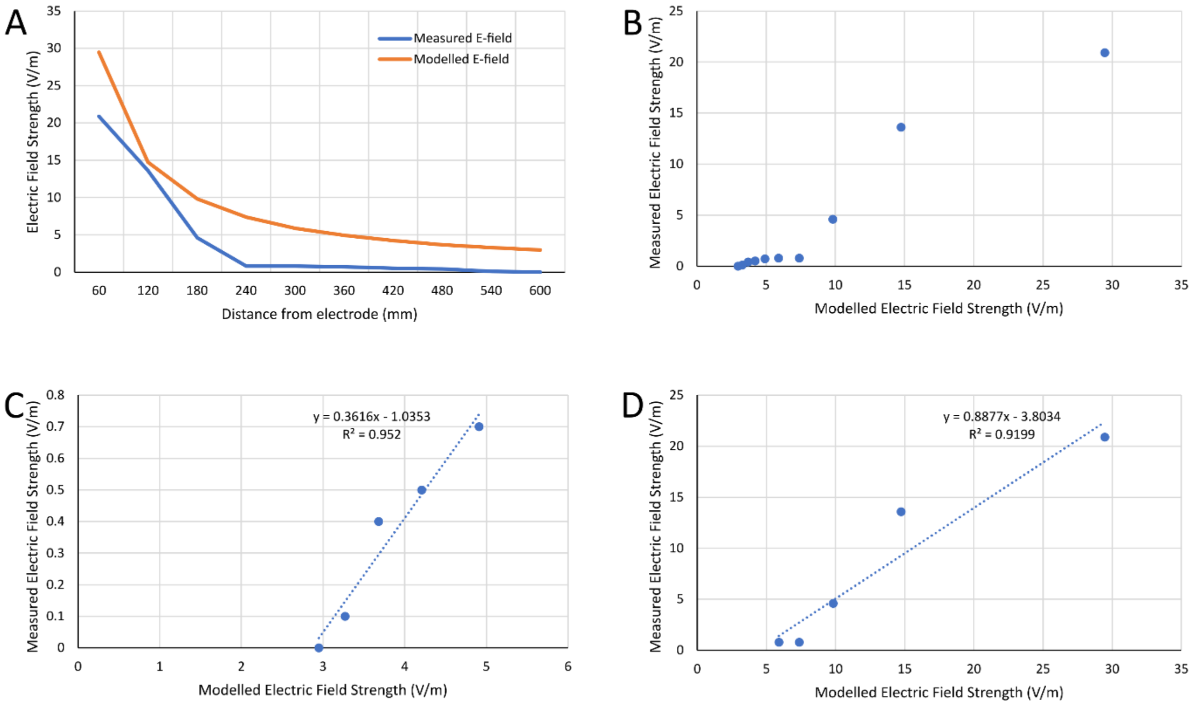

Figure S2. Relationship between measured and modelled experimental electric field strengths, Related to Figure 5. Panel A shows the measured and modelled electric field strengths produced by the stimulus electrode as a function of distance from the electrode. Panels B-D show the relationship between the measured and model E-fields, B demonstrates that there is not a linear relationship between the data points across all distances, however, dividing the data into far and near groups (C and D) reveals strong positive linear correlations.

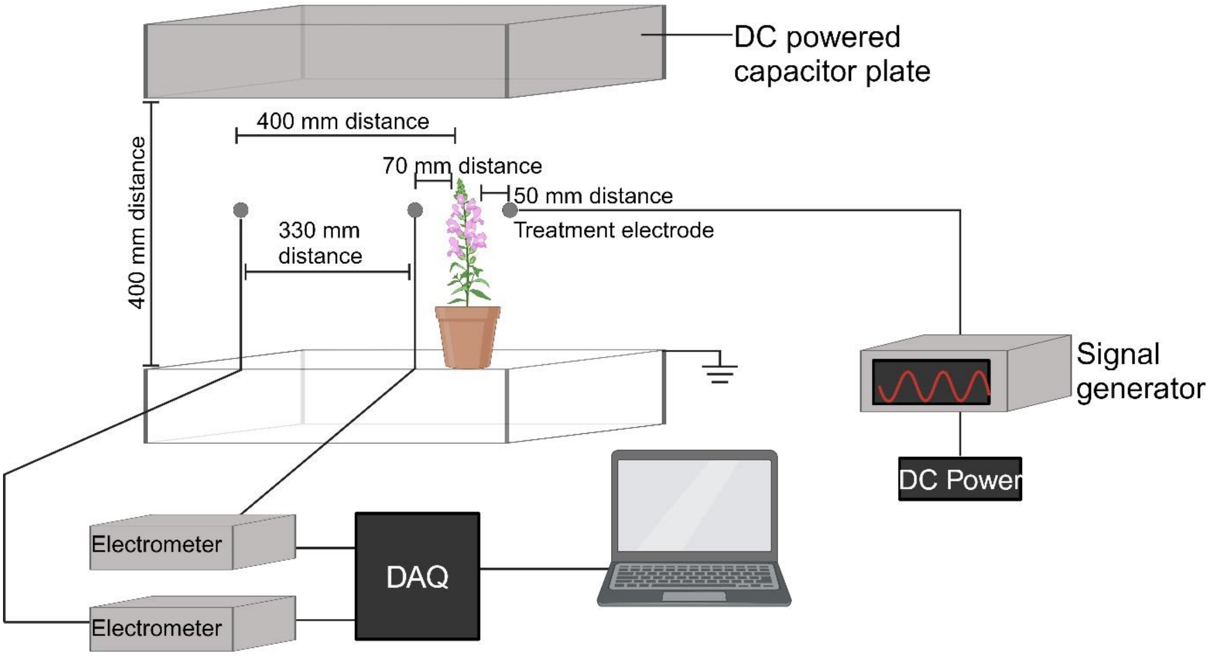

Figure S3. Laboratory electric field setup, related to Figure 5.

A schematic of the laboratory set-up used to measure experimental electric field strengths. Flower icon is for graphical illustration only and not to scale. Made using BioRender.com
